# Supplementary material for: Differential Effects of Gold Nanoparticles and Ionizing Radiation on Cell Motility between Primary Human Colonic and Melanocytic Cells and Their Cancerous Counterparts
Source: Int J Mol Sci. 2021 Jan 31;22(3):1418. doi: 10.3390/ijms22031418 (PMC7866826; doi:10.3390/ijms22031418)
Supplement: Supplementary file 1 [file ijms-22-01418-s001.pdf]

International Journal of *Molecular Sciences*

MDPI

Supplementary Figure S1

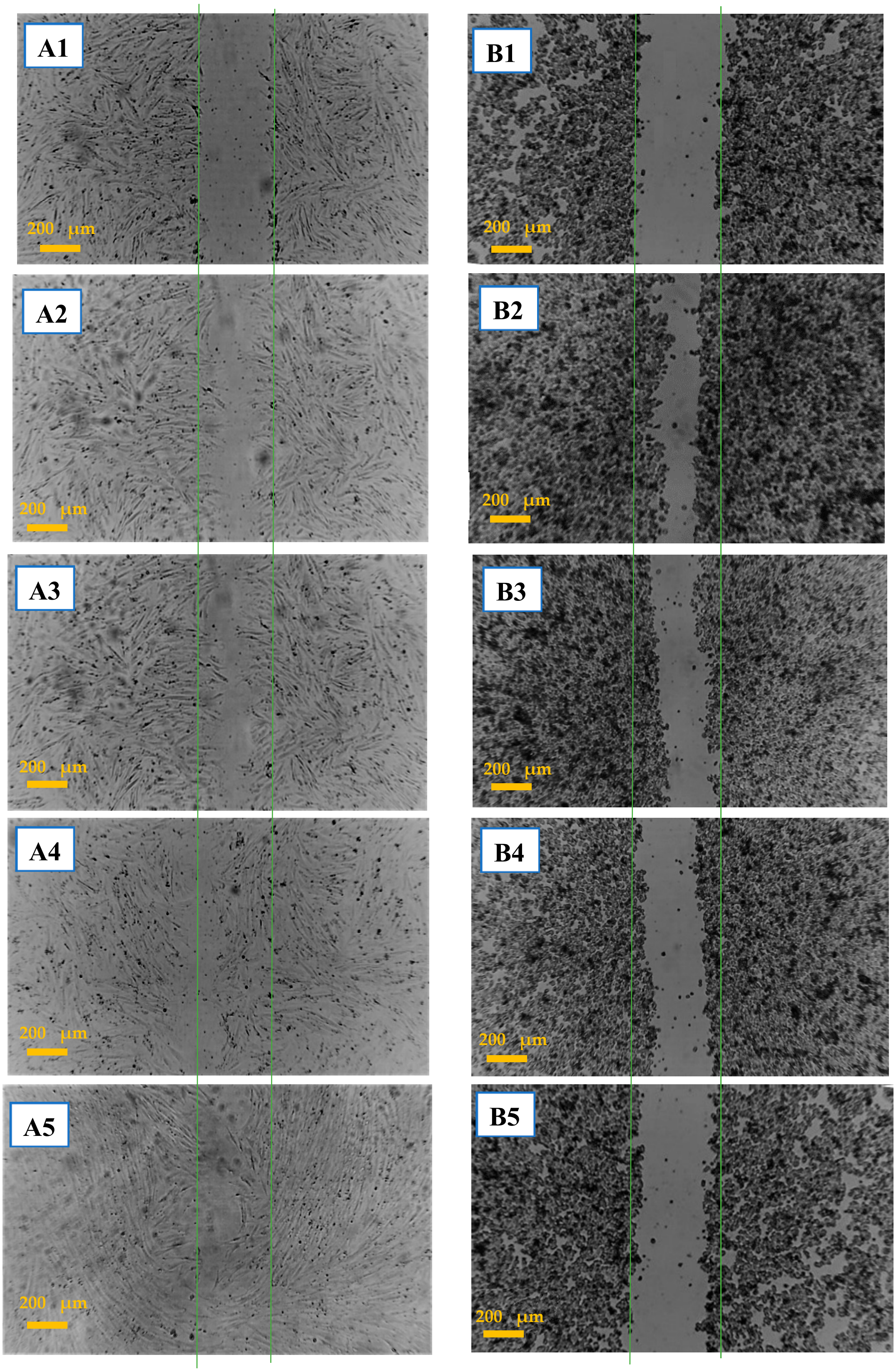

Figure 1: Effect of IR and/or AuNPs on the motility of human colonic cells. A) Human primary colon epithelial CCD841 cells; A1) untreated controls at 0 h, A2) untreated controls at 24 h, A3) 5 Gy irradiated cells at 24 h, A4) 1 mM AuNP-treated cells at 24 h and, A5) IR and AuNP-treated cells at 24 h and, B) human colorectal carcinoma SW48 cells; B1) untreated controls at 0 h, B2) untreated controls at 24 h, B3) 5 Gy irradiated cells at 24 h, B4) 1 mM AuNP-treated cells at 24 h and, B5) IR and AuNP-treated cells at 24 h.

Supplementary Figure S2

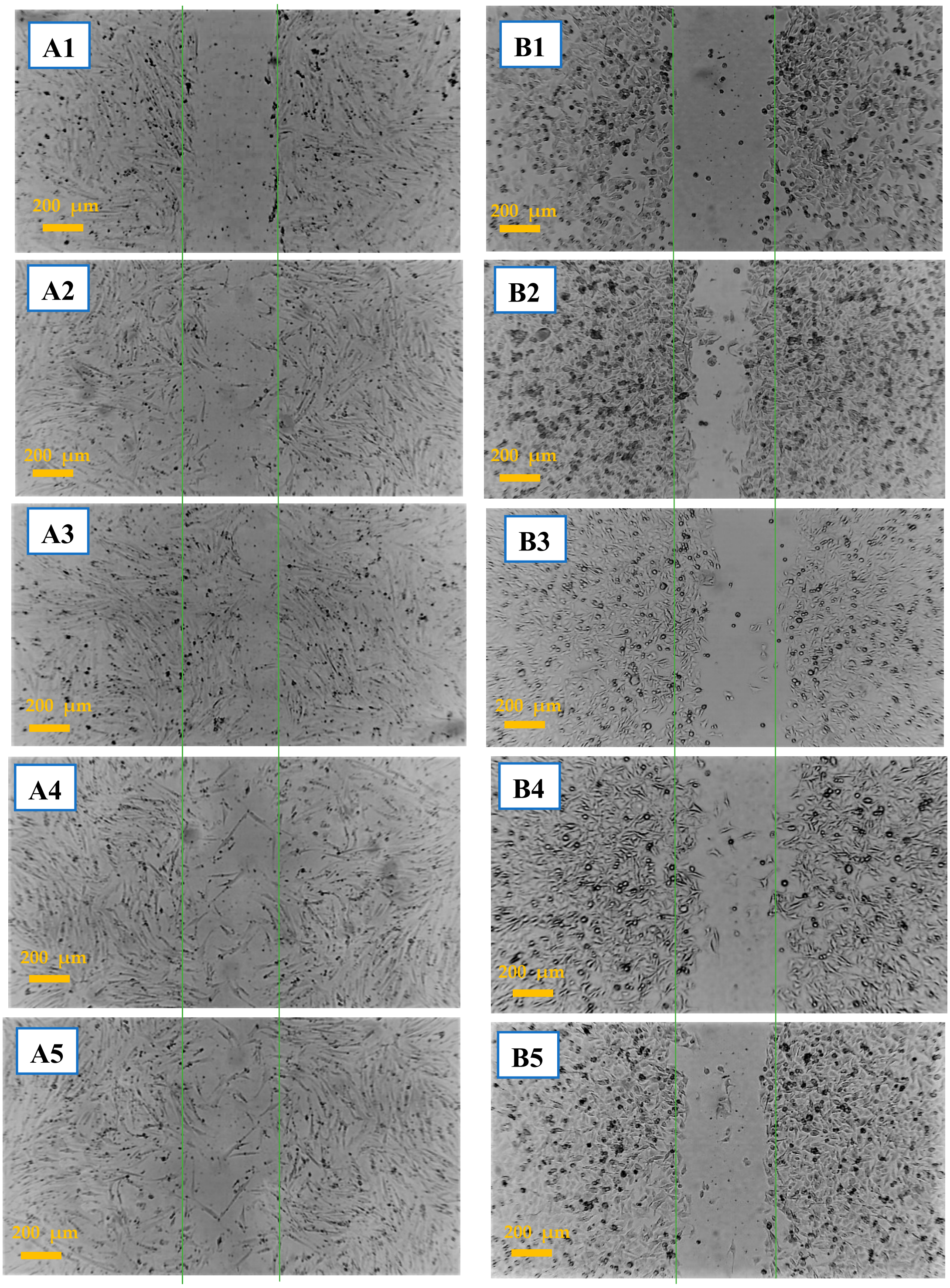

Figure 2: Effect of IR and/or AuNPs on the motility of human melanocytic cells. A) Human primary epidermal melanocytes; A1) untreated controls at 0 h, A2) untreated controls at 24 h, A3) 5 Gy irradiated cells at 24 h, A4) 1 mM AuNP-treated cells at 24 h and, A5) IR and AuNP-treated cells at 24 h and, B) human melanoma MM418-C1 cells; B1) untreated controls at 0 h, B2) untreated controls at 24 h, B3) 5 Gy irradiated cells at 24 h, B4) 1 mM AuNP-treated cells at 24 h and, B5) IR and AuNP-treated cells at 24 h.
